# Gene Expression Profiles and Flavonoid Accumulation during Salt Stress in *Ginkgo biloba* Seedlings

**DOI:** 10.3390/plants9091162

**Published:** 2020-09-08

**Authors:** Ningtao Xu, Sian Liu, Zhaogeng Lu, Siyu Pang, Lu Wang, Li Wang, Weixing Li

**Affiliations:** College of Horticulture and Plant Protection, Yangzhou University, Yangzhou 225009, China; Xu_ningtao0828@163.com (N.X.); 007243@yzu.edu.cn (S.L.); luzhaogeng@163.com (Z.L.); pangsiyu_0212@163.com (S.P.); wanglu971214@163.com (L.W.); liwang@yzu.edu.cn (L.W.)

**Keywords:** *Ginkgo biloba*, salt stress, flavonoid biosynthesis, gene expression

## Abstract

*Ginkgo biloba* is an economically valuable tree, as a variety of flavonoid compounds are produced by the leaves of its seedlings. Although soil salinity is a serious threat to agricultural productivity worldwide, the effect of salt stress on *G. biloba* seedlings remains unclear. In this study, we found that under high NaCl concentrations (200 and 300 mmol/L), seedling growth was inhibited and the water content, chlorophyll, and peroxidase (POD) enzyme activity were significantly decreased in the leaves, whereas the soluble protein and proline levels increased significantly. However, at low NaCl concentrations (50 and 100 mmol/L), the seedlings grew normally because of the regulation of catalase (CAT) and POD enzyme activities. To elucidate the molecular mechanisms behind *G. biloba* salt tolerance, we examined the transcriptome of *G. biloba* seedlings treated with 100 mmol/L NaCl. Twelve differentially expressed genes (DEGs) were found to be involved in ion osmotic potential signal transduction and amplification, including two ABA signaling genes, five CDPK/CIPK genes, and five mitogen-activated protein kinase (MAPK) signaling genes. We also found that NAC transcription factors may be involved in the salt stress response; these included positive regulators (*Gb_12203*, *Gb_27819*, *Gb_37720*, and *Gb_41540*) and negative regulators (*Gb_32549*, *Gb_35048*, and *Gb_37444*). Importantly, treatment with 100 mmol/L NaCl can significantly improve flavonoid and flavonol glycoside biosynthesis. Simultaneously, the expression of flavonoid biosynthesis-related genes, including *PAL* (*Gb_10949*, *Gb_21115*) and *FLS* (*Gb_00285*, *Gb_14024*, and *Gb_14029*), was significantly upregulated. Based on these results, we reveal that *G. biloba* seedlings can tolerate low-level soil salinity stress through the regulation of different kinds of genes and transcriptome factors, especially flavonoid biosynthesis, which is improved to respond to environmental stress.

## 1. Introduction

Soil salinity is one of the most significant environmental challenges currently threatening agricultural productivity, as it can retard plant growth and development [1]. Salt stress first affects the root system of plants, where it impairs growth by osmotic stress in the short term, then by salt-induced ion toxicity over the long term. Excessive levels of Cl^−^ and Na^+^ ions can disrupt the ability of plants to regulate Na^+^ and K^+^ uptake and cause other nutrient imbalances [2]. High levels of salt can also induce oxidative stress mediated by reactive oxygenic species (ROS), which disrupt regular lipid, protein, and nuclear metabolism [3,4]. Stress hormones such as abscisic acid (ABA), salicyclic acid (SA), jasmonic acid (JA), and ethylene, as well as growth hormones including auxin, cytokinins (CKs), gibberellic acid (GA), and brassinosteroids (BRs), which play important roles in mediating salinity stress signals and controlling the balance between growth and stress responses [5].

Salt stress can elicit extensive gene expression changes across physiological and molecular pathways, for example, in the transcription factors involved in ion transport regulation [6]. Under saline conditions, signal transduction begins with NaCl detection, and consequently, second messengers such as Ca^2+^, inositol phosphate, ROS, and phytohormones are generated. The salt-overly-sensitive (SOS) pathway has been studied extensively. Calcium is first detected by SOS3, which then binds SOS2; the SOS2-SOS3 complex can then phosphorylate the H^+^ antiporter SOS1, which transports sodium out of the cell [7]. Other genes with essential functions related to salt tolerance include cAMP-dependent protein kinases (CDPKs), the ABA signaling pathway, mitogen-activated protein kinase (MAPK) cascade genes, and the ethylene signal transduction pathway and its associated genes (e.g., *CTR1*, *EIN3*, and *ETR1*) [8,9]. Recently, the importance of transcription factor families, such as bZIP, MYC, WRKY, AP2, NAC, and DREB, in controlling gene expression in response to salt stress has been demonstrated [10].

*Ginkgo biloba* is an important economic tree; its leaves contain valuable flavonoids, which are used as an industrial raw material for the production of *G. biloba* extract (GbE) to treat human cardiovascular and cerebrovascular diseases [11]. Flavonoids are a major class of plant secondary metabolites, which play important roles in eliminating free radicals, preventing oxidation, and protecting plant growth and development [12]. Post-harvest *G. biloba* leaves that were treated with 200 mmol/L NaCl were found to significantly increase the accumulation of flavonoids [13]. In addition, flavonoid accumulation increased in *G. biloba* suspension cells following NaCl treatment [14]. Although only the leaves of *G. biloba* seedlings can be used to extract GbE, the effects of salt stress on the growth and flavonoid biosynthesis of *G. biloba* seedlings remains unexplored.

In our study, *G. biloba* seedlings were treated with different concentrations of NaCl. Through analyzing the physiological changes, we determined the salt tolerance of *G. biloba* seedlings. We further utilized transcriptomic responses to characterize the regulatory genes, and identified the important genes regulating flavonoids in *G. biloba*.

## 2. Results

### 2.1. Salt Treatment-Induced Physiological Changes in G. biloba Leaves

**Figure 1:**
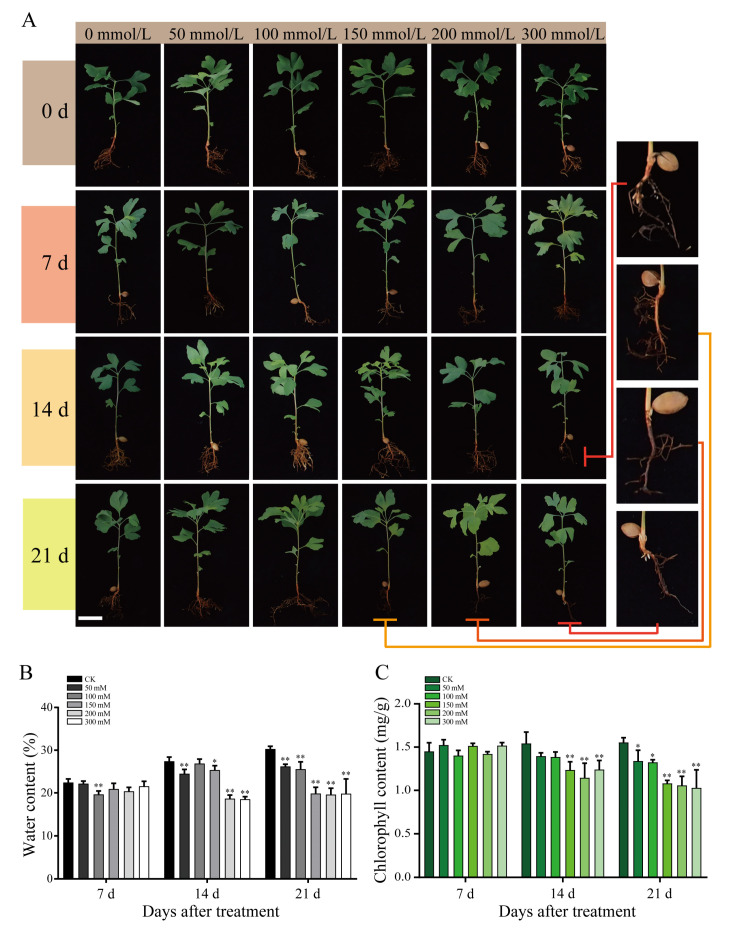
Growth of *G. biloba* seedlings. (**A**) Growth of *G. biloba* seedlings under 0, 50, 100, 150, 200, and 300 mmol/L NaCl concentrations harvested on days 0, 7, 14, and 21. The right insert shows an enlarged view of the roots treated with high NaCl concentrations. (**B**) Percentage water content of *G. biloba* seedlings. The vertical bars indicate standard deviation (SD; *n* = 16). (**C**) Total chlorophyll content of *G. biloba* seedlings. * *p* < 0.05; ** *p* < 0.01. The vertical bars indicate SD (*n* = 9).

Salt-treated *G. biloba* seedlings were sampled on days 0, 7, 14, and 21 for physiological observation (Figure 1). For plants treated with low NaCl concentrations (50 and 100 mmol/L), no significant physiological changes were detected. However, seedlings exposed to higher NaCl concentrations (200 mmol/L) exhibited significant atrophy of root systems on day 21. In particular, the root systems of seedlings treated with the highest NaCl concentration (300 mmol/L) began to atrophy significantly after 14 d. In addition, the water and chlorophyll contents of the seedlings also decreased significantly on days 14 and 21 at NaCl concentrations of 200 and 300 mmol/L (Figure 1).

**Figure 2:**
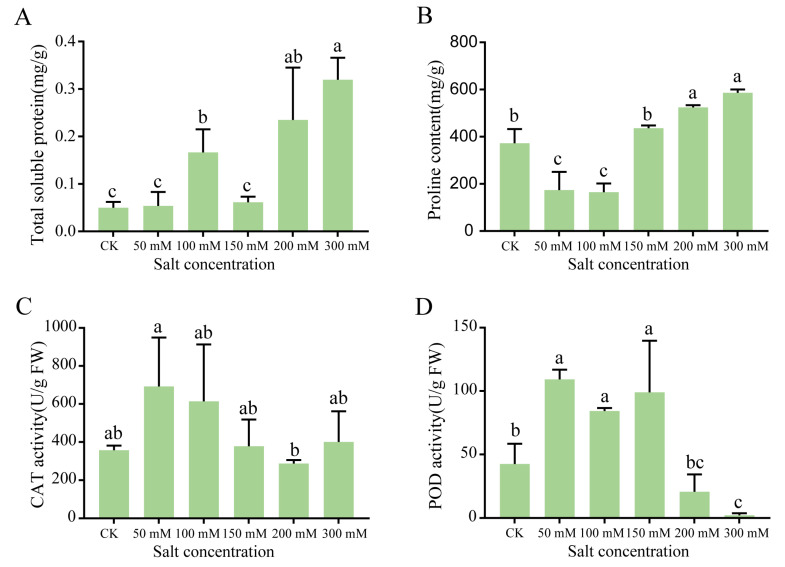
Physiological changes in *G. biloba* leaves after NaCl treatment. (**A**) Soluble protein content, (**B**) proline content, (**C**) catalase (CAT) activity, and (**D**) peroxidase (POD) activity. Samples were harvested after 14 days. Lower case letters represent significant differences (*p* < 0.05). The vertical bars indicate SD (*n* = 9).

To gain further insight into the effects of NaCl concentration on protein content and ROS stress, we analyzed the seedling samples harvested on day 14, given that the physiological effects of salt stress became obviously apparent after day 14 (Figure 1). The soluble protein content increased significantly as the NaCl concentration increased, except for 150 mmol/L salt concentration (Figure 2A). Relative to the control, the proline content decreased significantly under low NaCl concentrations (50 and 100 mmol/L) and increased significantly under higher concentrations (Figure 2B). The lowest proline content recorded was 165.11 mg/g at 100 mmol/L NaCl, which represented a 56% reduction in proline compared with the control. To investigate the oxygen free radical generation under salt stress, the catalase (CAT) and peroxidase (POD) activities in seedlings were then determined. The CAT activity was the highest at 50 mmol/L NaCl, reaching 629.27 nmol·g/min (Figure 2C). The POD activity increased significantly under low NaCl concentrations, but decreased significantly under high NaCl concentrations. At 50, 100, and 150 mmol/L NaCl, the POD activity reached 109.23, 84.17, and 98.97 nmol·g/min, respectively. At the higher NaCl concentrations of 200 and 300 mmol/L, POD activity decreased significantly to 20.63 and 2.24 nmol·g/min, respectively (Figure 2D).

### 2.2. Flavonoid Accumulation in G. biloba Leaves after NaCl Treatment

**Figure 3:**
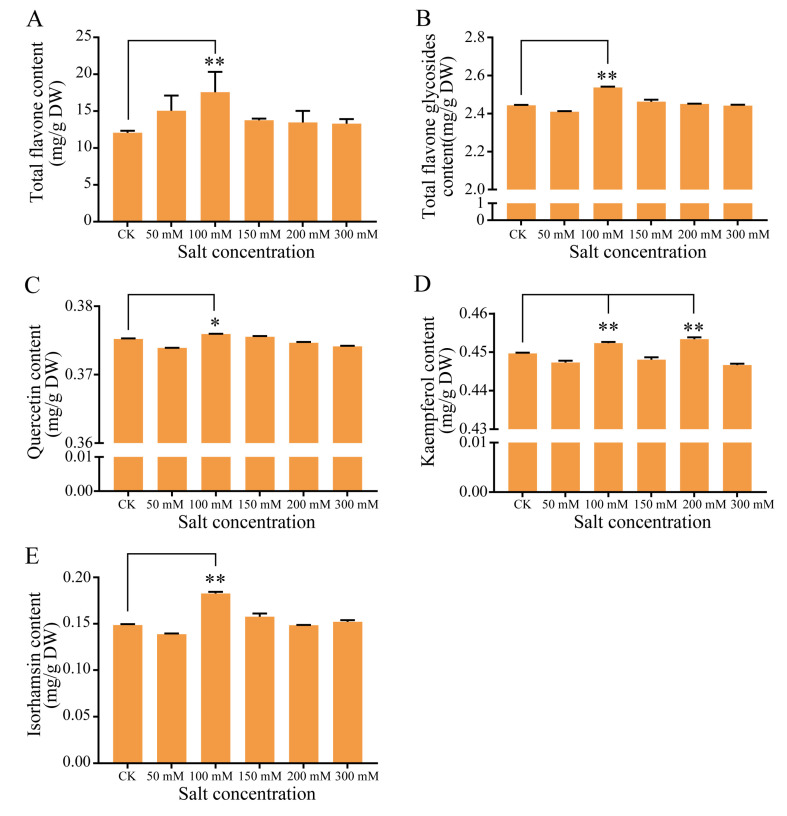
Total flavonoid and flavonol glycoside content in *G. biloba*. The contents of the (**A**) total flavones, (**B**) total flavone glycosides, (**C**) quercetin, (**D**) kaempferol, and (**E**) isorhamnetin were determined under 0, 50, 100, 150, 200, and 300 mmol/L NaCl conditions. The leaves were harvested after 14 days. * *p* < 0.05; ** *p* < 0.01. The vertical bars indicate SD (*n* = 9).

As flavonoids act as free radical scavengers and play an important role in resisting abiotic stress, we examined the flavonoid content of the leaves collected from seedlings grown under various salt concentrations for 14 days. The analysis revealed that the total flavonoid content was significantly higher under 100 mmol/L NaCl, while other treatments showed no significant difference compared with the control (Figure 3). Similarly, the total flavonol glycoside content was significantly higher at 100 mmol/L NaCl (2.54 mg/g) relative to the control (2.44 mg/g). We further characterized the flavonol glycoside contents, and discovered that the quercetin and isorhamnetin levels were significantly higher at 100 mmol/L NaCl than in the control. More specifically, the isorhamnetin content was 23% higher under the 100 mmol/L NaCl condition relative to the control. The kaempferol content also increased under the 100 mmol/L NaCl treatment condition (Figure 3).

### 2.3. Differential Gene Expression after NaCl Treatment

**Figure 4:**
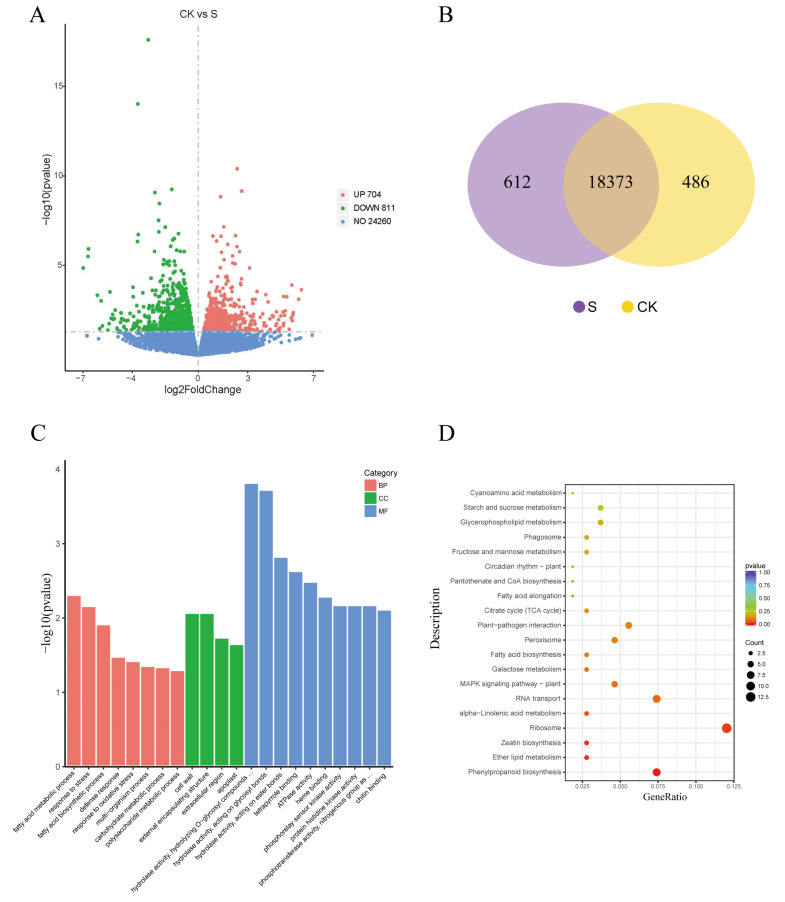
Differentially expressed genes (DEGs) and function enrichment. (**A**) Volcano plot of DEGs between 0 mmol/L NaCl (CK) and 100 mmol/L NaCl (S) leaves harvested after 14 days. (**B**) Venn diagram showing specific and overlapping transcripts identified in S and CK leaves. (**C**) Gene ontology (GO) functional classification of DEGs. BP—biological process; CC—cellular component; MF—molecular function. (**D**) Top 20 Kyoto Encyclopedia of Genes and Genomes (KEGG) pathways identified by the enrichment analysis. The vertical axis is the KEGG pathway, the horizontal axis is the ratio of the differentially expressed gene tree annotated to the KEGG pathway to the total number of differentially expressed genes.

Based on the phenotypic and physiological changes described above, we investigated the transcriptomic responses in the leaves of *G. biloba* seedlings grown under 100 mmol/L NaCl conditions. In total, 20.68 G and 20.56 G clean reads were obtained from the NaCl treatment and control group, respectively, of which more than 95% of the reads were mapped to the *G. biloba* genome. The gene expression patterns for both libraries were analyzed using the fragments per kilobase of exon model per million reads mapped (FPKM) method. The results showed that within the cDNA libraries, 18,985 and 18,859 genes were identified, and 612 and 486 genes were expressed specifically in NaCl-treated and control leaves, respectively. In total, 1515 differentially expressed genes (DEGs) were detected, including 811 upregulated and 704 downregulated DEGs in the leaves after NaCl treatment relative to the control (Figure 4). 

We then performed GO and KEGG enrichment analyses to investigate the biological functions of the DEGs. The GO enrichment analysis showed that 548 DEGs were enriched in several key GO terms, including response to stress (GO:0006950), defense response (GO:0006952), fatty acid biosynthetic process (GO:0006633), and carbohydrate metabolic process (GO:0005975). In addition, 108 DEGs were assigned to 65 KEGG pathways, of which phenylpropanoid biosynthesis, zeatin biosynthesis, and ether lipid metabolism pathways were the most significantly enriched. Other important pathways, including the MAPK signaling pathway, peroxisome, fatty acid biosynthesis, plant hormone signal transduction, and flavonoid biosynthesis, were also enriched (Figure 4).

### 2.4. Identification of Signaling Pathways Associated with Salt Stress

**Figure 5:**
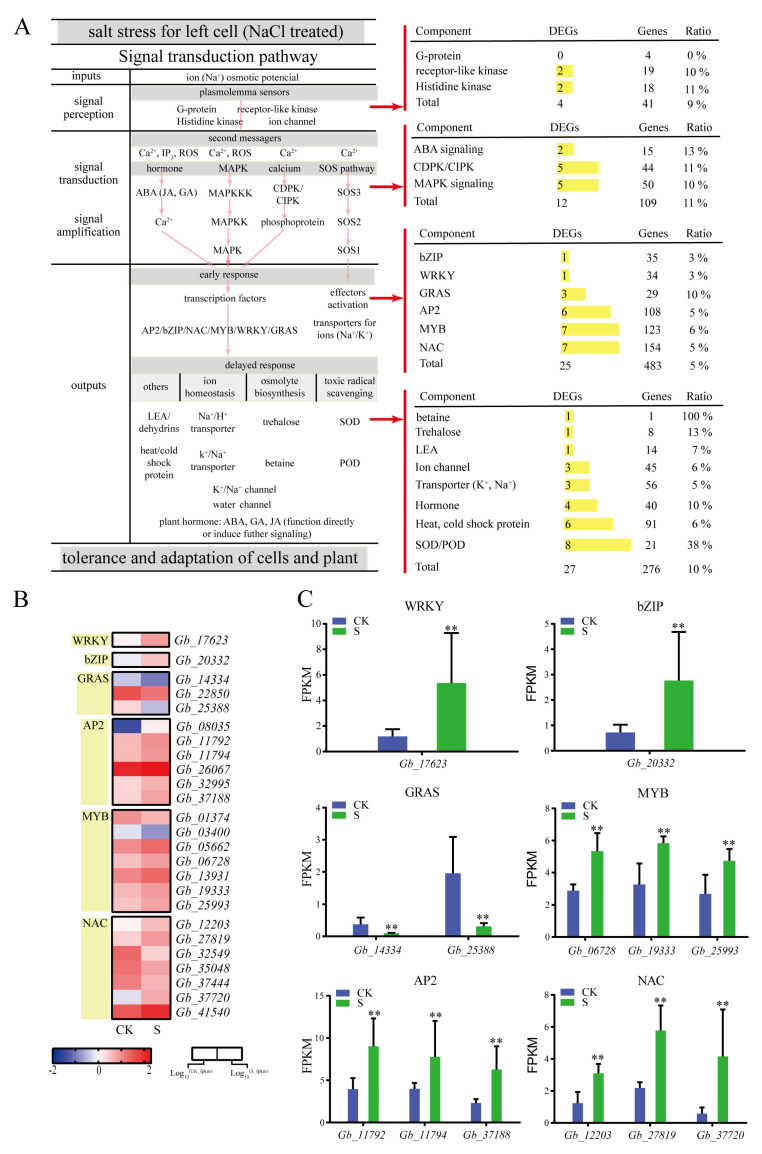
Expression of related genes under salt treatment. (**A**) Plant hormone, MAPK, calcium, and salt-overly-sensitive (SOS) pathways determine the framework of salt signaling. (**B**) Differentially expressed transcription factors (TFs) in *G. biloba* leaves following NaCl treatment. The fragments per kilobase of exon model per million reads mapped (FPKM) range from low (blue) to high (red) is –2 to 2, respectively. (**C**) Transcription factor genes with significantly different FPKM values. ** *p* < 0.01. The vertical bars indicate SD (*n* = 3).

Salt stress signal transduction pathways generally comprise the signal input (ion osmotic potential signal), signal perception, signal transduction and amplification, and signal output (Figure 5). Here, we identified 41 DEGs in *G. biloba* leaves encoding ion signal sensing-related proteins, including receptor-like kinase genes (*Gb_16715* and *Gb_32753*) and histidine kinase genes (*Gb_14997* and *Gb_05953*; Figure 5).

We also found 12 DEGs involved in ion osmotic potential signal transduction and amplification, including two ABA signaling genes, five CDPK/CIPK genes, and five MAPK signaling genes (Figure 5). In the MAPK signaling pathway, the negative regulator PP2C (*Gb_07154* and *Gb_30416*) of the ABA signal transduction pathway was significantly down-regulated. The early response genes associated with ion osmotic potential signaling included the following six transcription factor families: bZIP, WRKY, GRAS, AP2, MYB, and NAC (Figure 5). A total of 483 genes were identified as belonging to these six transcription factor families, 25 of which were differentially expressed under 100 mmol/L NaCl conditions (Figure 5). Among these transcription factors, MYB (*Gb_06728*), bZIP (*Gb_02332*), WRKY (*Gb_17623*), AP2 (*Gb_37188*), and NAC (*Gb_37720*) were significantly upregulated, the highest of which was NAC (*Gb_37720*), whose expression was six-fold higher than in the control. Of the delayed response genes, 276 genes, including 27 DEGs, were found to be associated with ion homeostasis, osmolyte biosynthesis, and toxic radicals. Of the effector factors, transporter (*Gb_08487*), trehalose (*Gb_07975*), and SOD/POD (*Gb_31013*) were significantly upregulated; the expression of SOD/POD (*Gb_31013*) was particularly high, displaying a 3.97-fold increase relative to the control (Figure 5).

### 2.5. Identification of Flavonoid Biosynthesis Genes Induced by Salt Stress

**Figure 6:**
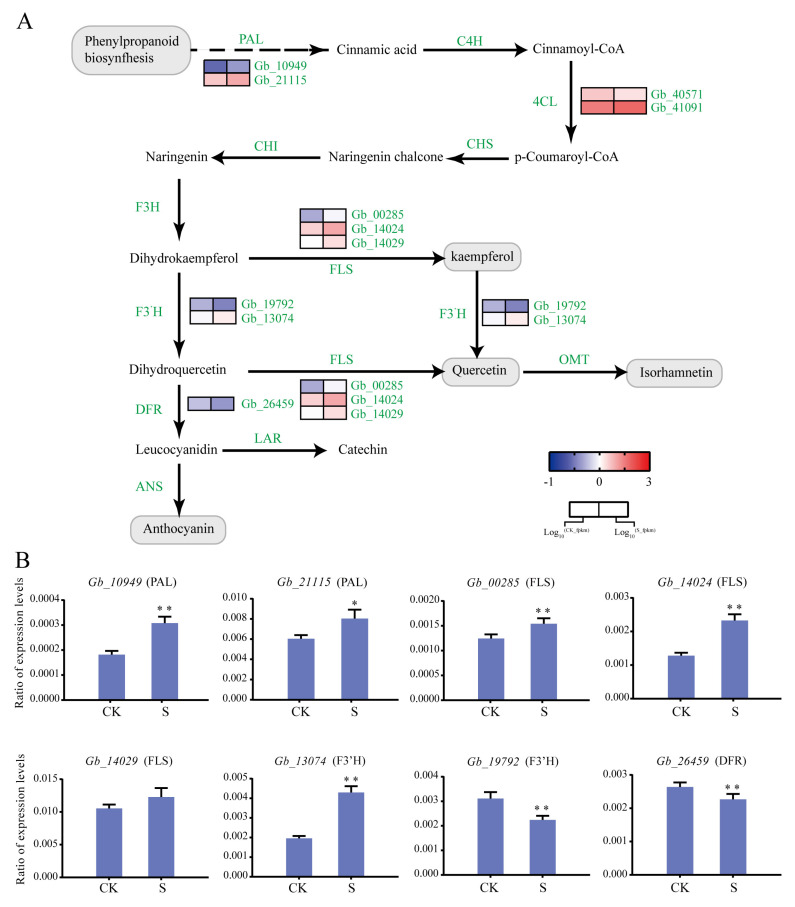
Differentially expressed structural genes involved in *G. biloba* flavonoid biosynthesis. (**A**) Expression profiles of DEGs involved in flavonoid biosynthesis after NaCl treatment. The FPKM range from low (blue) to high (red) is −1 to 3, respectively. (**B**) RT-qPCR analysis of eight flavonoid biosynthesis-related structural genes amplified from the RNA extracted from *G. biloba* seedling leaves grown under 0 and 100 mmol/L NaCl conditions. Leaves were harvested on day 14. * *p* < 0.05; ** *p* < 0.01. The vertical bars indicate SD (*n* = 3).

Analysis of the *G. biloba* flavonoid synthesis pathway identified 19 differentially expressed structural genes, including two *PAL*, two *4CL*, three *FLS*, two *F3′H*, and one *DFR* (Figure 6). Interestingly, most of the upstream genes in the flavonoid synthesis pathway were upregulated under 100 mmol/L NaCl treatment, such as *PAL* (*Gb_10949* and *Gb_21115*), *FLS* (*Gb_00285*, *Gb_14024*, and *Gb_14029*), and *F3′H* (*Gb_19792* and *Gb_13074*). However, the downstream gene DFR (*Gb_26459*) was downregulated. Additionally, *LAR* and *ANS* were not differentially expressed (Figure 6A). 

We further selected eight genes in the flavonoid pathway for the RT-qPCR analysis. We found that *PAL* (*Gb_10949* and *Gb_21115*) and *FLS* (*Gb_00285*, *Gb_14024*, and *Gb_14029*) were significantly upregulated. Of the *F3′H* genes, *Gb_13074* was upregulated and *Gb_19792* was downregulated. At the same time, *Gb_26459* and *Gb_06667* were downregulated, which is consistent with the RNA-seq results (Figure 6B).

## 3. Discussion

When plants are subjected to stress, they can increase their stress tolerance by regulating the production of certain metabolites that can reduce damage. Examples include synthesizing osmotic adjustment substances, such as proline, and soluble protein, as well as protective enzymes [15]. Salt stress can increase the osmotic potential and decrease soil water potential, which weakens the water absorption capacity of plant roots; reverse osmosis can even occur under physiological drought [16]. In this study, we found that under low concentration salt treatments, *G. biloba* seedlings could still grow normally following 21 days of treatment. Under high salt concentrations, root atrophy was apparent after 14 days, and significant decreases in the water and chlorophyll contents were also observed. Soluble protein and proline contents showed an overall upward trend as the salt concentration increased, whereas the CAT and POD activities followed a trend of first increasing and then decreasing with the increasing NaCl concentrations. These results indicated that *G. biloba* seedlings have some degree of salt tolerance, can grow for a relatively long time under low salt concentrations, and can also tolerate high salt concentrations for 14 days. 

Plant response to salt stress is a complex physiological process. When subjected to salt stress, plants will first respond at the gene level through transcriptional regulation, then RNA coding for salt stress-related proteins will be synthesized, after which a fine control of metabolite biosynthesis can be achieved to regulate plant metabolic and osmotic balance [17]. In our study, high-throughput transcriptome sequencing technology was used to analyze the *G. biloba* transcriptome after salt stress. Several proteins were identified to be signal perception, including G-protein with four genes but no DEGs, receptor-like kinase with 19 genes and two DEGs, and histidine kinase receptor protein with 18 genes and two DEGs. In addition, we identified two DEGs and 15 genes involved in ABA signaling, five DEGs and 44 genes involved in Ca^2+^ signaling, and five DEGs and 50 genes involved in MAPK signaling. Compared with other plant species [18], there were relatively few DEGs in the *G. biloba* seedlings after salt treatment, but the few DEGs that were identified were likely to be responsive to long-term low-salt stress.

Transcription factors in plants play major roles in regulating the expression of various inducible genes involved in plant growth and development, as well as adaptation to the environment [19]. In this study, several transcription factors responded significantly to salt stress, including the WRKY transcription factor, which has a highly conserved WRKY domain and has been reported to participate in responding to injury, aging, development, and disease [20]. In addition, WRKY is involved in the regulation of crop growth and development, and in stress responses. WRKY gene expression in *Thlaspi arvense* has been reported to be strongly induced by drought, NaCl, and low temperature [21]. NAC is a plant-specific transcription factor that plays an important role in plant biological and abiotic stress responses. Drought, salt, and low temperature can induce the *Arabidopsis* NAC transcription factor gene to participate in the abiotic stress response [22,23]. In this study, 25 DEGs and 483 genes expressed were identified in *G. biloba* leaves after salt treatment. WRKY (*Gb_17623*) significantly increased by eight-fold under salt treatment relative to the control. In addition, seven members of the NAC transcription factor family were differentially expressed between salt conditions; four of these genes (*Gb_12203*, *Gb_27819*, *Gb_37720*, and *Gb_41540*) were upregulated and three (*Gb_32549*, *Gb_35048*, and *Gb_37444*) were downregulated, suggesting that NAC transcription factors play important roles in the salt stress response of *G. biloba*.

Flavonoids are a class of secondary metabolites in plants and are widely found in vegetables, fruit trees, crops, and medicinal plants. Plants likely evolved to synthesize these valuable compounds for protection from pathogenic microorganisms and adverse environmental conditions [24,25]. The flavonoids in *G. biloba* are the main physiologically active ingredients, which play an important role in eliminating free radicals and protecting plant growth and development [26]. In the pathways that govern flavonoid biosynthesis, many genes showed a significant upregulation, including *PAL*, *4CL*, *FLS*, and *F3′H* genes, and *DFR* gene. These significantly upregulated, highly-expressed genes may contribute to the accumulation of flavonoids [27]. Flavonoids can enhance the ability of plants to resist drought and high salt stress. Recent studies have found that there is also a close relationship between flavonoids and salt stress. Walia et al. [28] compared the expression profiles of two different rice varieties grown under salt stress, and found that the flavonoid biosynthetic pathway was induced in the salt-sensitive strain IR29; the *PAL*, *CHS*, *CHI*, *F3H*, and *DFR* transcripts were all upregulated. Wahid and Ghazanfar [29] found that the contents of soluble polyphenols, anthocyanins, and flavonoids of salt-tolerant and salt-sensitive sugarcane lines increased under salt stress; the salt-tolerant strains exhibited 2.5-, 2.8-, and 3.0-fold higher levels of soluble polyphenols, anthocyanins, and flavonoids, respectively, when compared to a salt-sensitive strain. In the present work, we identified 19 differentially expressed structural genes in the flavonoid synthesis pathway in *G. biloba* leaves; of particular significance, the expression levels of two *PAL*, two *4CL*, three *FLS*, and two *F3′H* genes were upregulated after salt treatment. RT-qPCR analysis confirmed that the expression profiles of these genes were similar to those observed using RNA-seq. Consistent with the transcriptome profiling results, the content of flavonoids and flavonol glycosides increased significantly after treatment with 100 mmol/L NaCl in *G. biloba* seedings, which was different from those treated with 200 mmol/L NaCl in post-harvest *G. biloba* leaves. Collectively, these results suggest that long-term culturing with low-level salt treatment could facilitate flavonoid accumulation in *G. biloba* leaves.

## 4. Materials and Methods

### 4.1. Plant Materials and NaCl Treatment

*Ginkgo biloba* seedlings were grown in a greenhouse at 25 °C for 3 months. High-quality *G. biloba* seedlings were selected and divided into six groups, with 16 pots for each group. According to previous results [13,14], we set the NaCl concentrations at 0 (control) 50, 100, 150, 200, and 300 mmol/L NaCl solution. According to the method of Liu Airong [30], plants were watered with 500 mL of each NaCl concentration at 7-day intervals. The experimental treatment process was concluded after 21 days. At 0, 7, 14, and 21 days of NaCl treatment, approximately 10 g of leaves were collected from the same part of each treated plant for water content and chlorophyll content determination.

### 4.2. Determination of Flavonoid and Flavonol Content

For the spectrophotometric analysis, the flavonoids were extracted using a plant flavonoid kit (Suzhou Comin Biotechnology Co. LTD, Suzhou, China), according to the manufacturer’s instructions. Samples were dried to a constant weight and passed through a 40-mesh sieve. Approximately 0.02 g of the crushed plant material was added to 2 mL of 60% ethanol, and incubated with oscillation at 60 °C for 2 h. The samples were then centrifuged at 10,000× *g* for 10 min at 25 °C. The supernatant was collected and incubated at 25 °C for 15 min, then the light absorption value at 510 nm was determined.

For high-performance liquid chromatography (HPLC) analysis of the flavonoids, the samples were dried to a constant weight and passed through an 80-mesh sieve. The powder (0.2 g) was added to 2 mL of 60% alcohol in a centrifuge tube, followed by ultrasonic extraction for 20 minutes to extract the supernatant, and was repeated three times. Samples were centrifuged at 10,000× *g* at 4 °C for 10 min and the supernatant collected. Approximately 1 mL of the supernatant was absorbed and filtered through a 0.22-μm membrane. The content of flavonol glycosides in *G. biloba* was determined by HPLC-mass spectrometry (HPLC-MS) using an Agilent Eclipse Plus C18 column (150 × 12 mm, 5 μm, Agilent, Shanghai, China). The solvents used for separation were acetonitrile (solvent A) and water containing 0.1% methanol (solvent B). Gradient elution was performed as follows: 0–12 min (20% A + 80% B), 12–13 min (60% A + 40% B), 13–16 min (100% A), and 16–20 min (20% A + 80% B). The following settings were applied: flow rate, 0.3 mL/min; column temperature, 35 °C; injection volume, 10 μL; UV detector, 370 nm. In addition, HPLCI-MS adopts a negative ion mode, and mass acquisition is the MRM mode. Quercetin (SQ8030, Solarbio, Beijing, China), isorhamnetin (SI8280, Solarbio), and kaempferol (SK8050, Solarbio) were included as standards.

### 4.3. Detection of ROS Metabolism

Changes in ROS metabolism in *G. biloba* under salt stress were detected using a soluble protein kit, proline (Pro) kit, catalase (CAT) kit, and peroxidase (POD) kit (Suzhou Comin biotechnology Co., LTD) according the manufacturer’s instructions in each case.

### 4.4. Transcriptome Sequencing

Based on the flavonol content results, six libraries (CK (0 mmol/L NaCl solution) and S (100 mmol/L NaCl solution) groups at day 14, three biological replicates per group) were prepared for RNA-seq. Raw data (raw reads) of fastq format were firstly processed through in-house perl scripts. In this step, clean data (clean reads) were obtained by removing reads containing adapter, reads containing ploy-N, and low-quality reads from the raw data. The quality scores (Q20 and Q30) and GC contents of the clean data were calculated. The FPKM of each gene was calculated according to its length, and the gene expression level was estimated.

### 4.5. Identification and Functional Analysis of Differentially Expressed Genes

DESeq2 software (1.16.1) was used for analysis of the differential expression between the two NaCl concentrations selected for comparison (three biological replicates per group). A corrected *p*-value of 0.05 and an absolute fold change of 0 were set as the thresholds for significant differential expression. Gene ontology (GO) enrichment analysis of DEGs was performed using cluster Profiler (3.4.4) software [31], which corrected for gene length bias. GO terms with corrected *p*-values < 0.05 were considered to be significantly enriched. Statistical enrichment of differentially expressed genes in the Kyoto Encyclopedia of Genes and Genomes (KEGG) pathway were analyzed using cluster Profiler (3.4.4).

### 4.6. Real-Time Quantitative PCR (RT-qPCR)

The total RNA was extracted from the *G. biloba* leaves treated with water or 100 mmol/L NaCl solution using an RNAprep Pure Plus Kit (TIANGEN, Beijing, China), according to the manufacturer’s instructions. Reverse transcription of RNA into cDNA was performed using the Prime Script RT reagent Kit (TAKARA, Beijing, China) with corresponding primers. Primers for RT-qPCR were designed using Primer 5.0 software (Premier Biosoft, San Francisco, CA, USA), and are listed in Table 1. Primers for the *G. biloba GAPDH* gene (GenBank, landing number: L26924) were included as an internal control. RT-qPCR reactions were executed using a CFX96 Real-Time System (BioRad, Hercules, CA, USA); the reaction mixture and thermocycling conditions followed a previously described method [32].

## 5. Conclusions

**Figure 7:**
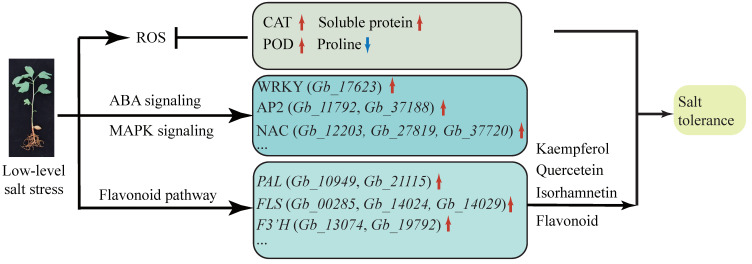
Model diagram of signal pathway and metabolic pathway changes in *G. biloba* seedings under low-level salt stress.

*Ginkgo biloba* exhibits a certain degree of salt tolerance, which permits it to grow normally in 100 mmol/L NaCl. The content of flavonoids in its leaves is also increased in response to NaCl. By transcriptome analysis, we identified 12 DEGs involved in ion osmotic potential signal transduction, particularly NAC transcription factors that may be involved in salt stress. In the flavonoid pathway, the *PAL* (*Gb_10949* and *Gb_21115*) and *FLS* (*Gb_00285*, *Gb_14024*, and *Gb_14029*) genes maintain high levels of expression under salt stress, indicating that long-term salt treatment can promote the biosynthetic metabolism of flavonoids through *PAL* and *FLS* (Figure 7).

## Figures and Tables

**Table 1:** Primers used for a subset of DEGs in this study.

Gene ID	Forward Primer (5′-3′)	Reverse Primer (5′-3′)
Gb_14024	CTTGCTCTCGGCGTCGAATCTC	ACTTGGAGTCCTGGCACATCATTG
Gb_14029	TCCTGAGCAACGGCAAGTTCAAG	GAATACCGGCCACGACATCCTTAC
Gb_00285	TGGTGGATGAAGGCATTGACAGC	ACAACAGCAGATCGGCGTTCAC
Gb_06667	GCCCTCAGAATGGGAACGGA	AGCATCTGAAGCGAGGCCTT
Gb_13074	GTCGTCACAGATAGCCGCTTGG	AGTGTCTCCTTGGCAACGCAATC
Gb_21115	AGGAAGGGCTGGCTTTGGTAAATG	TCGCAGAACATAGCAGACGCAATC
Gb_10949	AATGTGCTTCCTGTGCTGTCTGAG	ATCCTGCTTGGGTTTGGGTTTCTG
Gb_26459	TGAGTCGCAGGACCCAGAGAAC	CGCTTGACAGACTTTGCCTTTGC
Gb_19792	GTGTGGTGCCGAGCTTGAGATAC	ATGAGGAGGAGGAAGAGGTTGCC
